# Formative research to inform the development of a peer-led HIV self-testing intervention to improve HIV testing uptake and linkage to HIV care among adolescents, young people and adult men in Kasensero fishing community, Rakai, Uganda: a qualitative study

**DOI:** 10.1186/s12889-020-09714-1

**Published:** 2020-10-20

**Authors:** Joseph K. B. Matovu, Aminah Nambuusi, Scovia Nakabirye, Rhoda K. Wanyenze, David Serwadda

**Affiliations:** 1 Department of Disease Control and Environmental Health, Makerere University School of Public Health, P.O. Box 7072, Kampala, Uganda; 2 Busitema University Faculty of Health Sciences, Mbale, Uganda

**Keywords:** Peer-led, HIV, Self-testing, Fishing community

## Abstract

**Background:**

Despite efforts to improve HIV testing and linkage to HIV care among adolescents, young people and adult men, uptake rates remain below global targets. We conducted formative research to generate data necessary to inform the design of a peer-led HIV self-testing (HIVST) intervention intended to improve HIV testing uptake and linkage to HIV care in Kasensero fishing community in rural Uganda.

**Methods:**

This qualitative study was conducted in three study communities in Kasensero fishing community in Rakai district, Uganda, in May 2019. Six single-sex focus group discussions (FGDs) comprising 7–8 participants were conducted with adolescents and young people (15–24 years) and adult men (25+ years). We collected data on people’s perceptions about peer-led HIVST; potential acceptability of a peer-led HIVST intervention and suggestions on how to improve linkage to HIV care after a positive HIVST result. Peer-led HIVST was defined as an approach where trained lay people distribute HIVST kits to other people in the community. FGDs were audio-recorded with permission from the participants, transcribed verbatim and analysed manually following a thematic framework approach.

**Results:**

Forty-seven participants (31 men and 16 women) participated in the FGDs. Across communities and age-groups, most participants mentioned that peer-led HIVST would be generally acceptable to people in the fishing community but people will need support in performing the test due to fear of performing the test wrongly or failing to cope with HIV-positive results. Most participants felt that peer-led HIVST would bring HIV testing services closer to the community “*because [the peer-leader] could be my immediate neighbour*”, making it easier for people to obtain the kits at any time of their convenience. To improve linkage to HIV care, participants felt that the use of peer-leaders to deliver the initial ART dose to self-tested HIV-positive individuals would be more preferable to the use of community-based ART groups or home-based ART initiation.

**Conclusion:**

Our study shows that peer-led HIVST is potentially acceptable in the fishing community. These findings suggest that this approach can improve uptake of HIV testing and linkage to HIV care services among populations that are usually missed through conventional HIV testing services.

**Supplementary information:**

**Supplementary information** accompanies this paper at 10.1186/s12889-020-09714-1.

## Background

Reaching adolescents, young people and adult men with HIV testing services is critical for the attainment of the first “95%” of the UNAIDS 2030 global targets dubbed 95–95-95: that is, by 2030, 95% of all people living with HIV will know their HIV status; 95% of all people with diagnosed HIV infection will receive sustained antiretroviral therapy; and 95% of all people receiving antiretroviral therapy will have viral suppression [1]. However, uptake of HIV testing services and subsequent linkage to HIV care among adolescents, young people and adult men remain critically below threshold levels particularly in sub-Saharan Africa (SSA) [2]. Studies show that individuals aged 15–24 years are less likely to be aware of their HIV status, to be enrolled in HIV care and to have a suppressed viral load when compared to HIV-positive persons aged 30 years or older [3, 4]. For instance, results from population-based HIV impact assessments in Malawi, Zambia and Zimbabwe show that 48% of adolescents and young people (15–24 years) living with HIV were aware of their HIV status in 2017 compared to 65% of those aged 25–34 years and 78% of those aged 35–59 years [5]. This means that majority of the adolescents and young people living with HIV remain unaware of their HIV status, despite the fact that nearly one-third of new HIV infections globally occur among this age-group [1].

Several barriers continue to inhibit adolescents and young people from utilizing HIV testing services including low HIV risk-perception [6], long waiting times at HIV testing clinics [7], concerns around confidentiality of HIV test results by health workers [8], lack of youth-friendly services [9], and fear of knowing their HIV status [10, 11]. Similar barriers continue to inhibit HIV testing uptake among adult men, including fear for testing positive for HIV, stigma associated with HIV, and fear that their HIV-positive results may not be kept confidential by health workers [12], and call for a need to design interventions that can reduce these barriers in order to improve HIV testing coverage and linkage to HIV care among adolescents, young people and adult men who are often missed in HIV testing and treatment programs [13, 14].

However, while previous interventions aimed at increasing HIV testing among men have been relatively successful [15–19], uptake of HIV testing services among men remains generally below the 90% threshold. In Rakai district, south-western Uganda, for instance, Billioux, Chang, Reynolds, et al. [4] found that men were 25% less likely to engage in HIV care than women, with men living in high-risk fishing communities significantly less likely to enroll in HIV care than men in other communities. Findings from the 2016 Uganda Population-based HIV Impact Assessment (UPHIA) confirm results from previous studies showing that men are less likely to test for HIV and, if HIV-positive, to link to HIV care than women. For instance, only 39.7% of men aged 15–19 years had ever tested for HIV and received their HIV test results compared to 53.8% among women of the same age. Among those aged 20–24 years, 73.3% of men reported that they had ever tested for HIV and received their HIV test results compared to 91.9% among women of the same age [20]. Findings from the same report also show that 75.8% of young HIV-positive men aged 20–24 years were unaware of their HIV sero-positive status compared to 54.2% of young HIV-positive women of the same age. Among people living with HIV who knew their HIV status, only 18% of young HIV-positive men aged 20–24 years were on ART compared to 41.5% of young HIV-positive women of the same age. Hegemonic masculinity norms that deter men from accessing health facility-based HIV services [21–23] have been implicated as the leading cause of men’s low uptake of HIV testing and treatment services, particularly those provided at health facilities. Evidence shows that men visit primary care facilities less frequently than women, have fewer preventive health checks and when they seek medical help, ask fewer questions than women [2, 5]. It is probably for this reason that UNAIDS, in 2017, dubbed boys and men as the ‘blind spot’ in the HIV response [2].

Recent evidence suggests that a network-based strategy can improve HIV testing and linkage to HIV care particularly among men who have sex with men testing for the first time [24–26]. Using a social network-based strategy to distribute HIVST kits to African American and Latino MSM, Lightfoot, Campbell, Moss, et al. [24] found that individuals reached through a peer-based HIVST strategy were significantly more likely to have never tested for HIV than MSM reached through community-based HIV testing programs. Similarly, Lippman, Lane, Rabede, et al. [25] found that network distribution of HIVST kits not only reached first-time MSM testers but also increased the frequency of HIV testing from 37.8% before to 84.5% after introduction of HIVST. Tun, Vu, Dirisu et al. [26] reported 100% linkage to HIV care among HIV-positive MSM who were identified through an intervention that distributed HIVST through key opinion leaders. Taken together, these findings suggest that network-based distribution of HIVST can improve HIV testing coverage, particularly in hard-to-reach and largely mobile populations, including people living in the fishing communities. However, few such studies have assessed the effect of social network-based interventions in improving uptake of HIV services among key and priority populations in sub-Saharan Africa [27, 28]. Our study extends social network-based research by focusing on the use of peer-leaders to distribute HIV self-test kits within existing social networks in a fishing community setting. Fishing communities present unique challenges: they are located in remote areas, far away from main health services, have high HIV prevalence and their populations are largely mobile [29], which complicates access to conventional HIV services. Innovative interventions are therefore needed to reach people living in such settings with HIV interventions.

At the moment, little evidence exists on how best such interventions can be designed to reach people living in highly mobile and remote fishing communities. The objective of this study was to explore adolescents, young people and adult men’s perceptions about the potential acceptability of a peer-led HIV self-testing intervention to generate the evidence needed to inform the design and implementation of a pilot, social network-based, peer-led HIV self-testing intervention intended to improve HIV testing uptake and linkage to HIV care in Kasensero fishing community in rural Uganda.

## Methods

### Study site

The study was conducted in Kasensero, a hyperendemic fishing community, along the shores of Lake Victoria in Rakai district. Kasensero fishing community has the highest HIV prevalence in Uganda with average adult HIV prevalence ranging between 37 and 41% [30–33]. HIV prevalence was 42.5% among females and 31.7% among males in 2017 [30]. The fishing community is composed of three study communities, namely: Kasensero landing site, Gwanda and Kyebe. Almost 90% of the residents of Kasensero landing site are engaged in fishing or fishing-related activities (e.g. net repairing, boat making/repairing; fish drying and fish vending) although people engage in other occupations including farming, *boda-boda* motorcycle riding (motorcycle taxis), operating restaurants, and engaging in commercial/business activities, among others, particularly as one moves away from the landing site [34]. The fisher-folk population is unique in multiple respects: they tend to migrate between landing sites in search of fish as the fish season changes; and the fishermen tend to spend a number of days on the lake which denies them access to health services, including HIV testing and linkage to HIV care [35]. Besides, the fishing crews generally work during the night hours and collect the fish in the early morning. Thus, if they are not fishing, some of them tend to be idle during the day and may spend time consuming alcohol or engaging in sexual activity since there are few recreational services on the Lake shores.

At the time of the study, HIV self-test kits were only available at the existing health facilities for distribution to key populations and male partners of pregnant women accessing antenatal care services at those health facilities as part of the government strategy to improve HIV testing among key populations and male partners of pregnant women. This service was introduced to the study community in November 2018 by the Rakai Health Sciences Program; a research organization that conducts health research in Rakai district, including in Kasensero fishing community. Our study aimed to explore if delivery of HIVST kits through existing social networks in the community, could be acceptable as an alternative HIVST distribution mechanism to increase HIV testing and linkage to HIV care (among those testing HIV-positive) in this fishing community.

### Study design

This was a qualitative study that was conducted as part of a large study intended to assess the effect of a peer-led HIV self-testing intervention on HIV testing uptake and linkage to HIV care among adolescents (15–19 years), young people (20–24 years) and adult men (25+ years) in Kasensero fishing community.

### Participants selection

Study participants were purposively selected, working closely with community health mobilizers (who are trained lay people who serve as health volunteers in their respective communities). We obtained lists of trained community health mobilizers from the Rakai Health Sciences Program. A staff of the Rakai Health Sciences Program helped to introduce members of the study team to at least one community health mobilizer in each study community who then introduced them to other community health mobilizers in the community. A week to the focus group discussion (FGD), the study team contacted community health mobilizers in each community and asked them to identify a venue for the FGD and invite up to eight participants to attend the FGD on a pre-determined date. Community health mobilizers were asked to identify participants, stratified by age-group into adolescent boys and young men (15–24 years), adolescent boys and young women (15–24 years) and adult men (25+ years), depending on the type of FGD that had been allocated to a particular community (see Table 1). Because we intended to gain insight into how many social network groups existed in the community, we asked the community health mobilizers to ensure that each FGD included people who belonged to as many social network groups as those that they knew themselves.

**Table 1:** Composition of FGDs conducted in Kasensero fishing community

FGD No.	Study Community	Participants’ characteristics	Group composition
1	Kasensero	Adult men (25+ years)	Eight participants: six (6) were fishermen, one (1) was a boat pusher while the remaining participant was a boat engineer
2	Kasensero	Adolescent girls and young women (15–24 years)	Eight participants: one (1) was a hair dresser, one (1) was a sex worker, one (1) was a vegetable vendor, one (1) was a drug shop attendant, one (1) was a tailor while three (3) were house wives.
3	Gwanda	Adolescent boys and young men (15–24 years)	Eight participants: four (4) were boda-boda cyclists, one (1) was a student, one (1) was a bricklayer, one (1) was a motorcycle mechanic and one (1) was a farmer
4	Gwanda	Adolescent girls and young women (15–24 years)	Eight participants: six were trainees in the DREAMS project and two were housewives
5	Kyebe	Adult men (25+ years)	Seven participants: one (1) was a casual labourer, one (1) was a Security Guard while five (5) were farmers.
6	Kyebe	Adolescent boys and young men (15–24 years)	Eight participants: five (5) were boda-boda cyclists, two (2) were farmers and one (1) was a shopkeeper

We used the term ‘social network group’ in the generic sense to refer to any social groupings of people in the community who worked together, or did the same job (e.g. fishing) or met more often and who would be considered close friends or close associates. Since the purpose of the planned peer-led HIV self-testing intervention was to use social network members to reach other members in their networks, it was important that we obtained perspectives on HIV self-testing from different social network groupings to inform the design of the intervention. It was not necessary for community health mobilizers to select participants that they knew; the most important issue was the recruitment of participants representing different social network groupings. During the selection of the participants, community health mobilizers worked as individuals, and approached people (including those that they did not know personally) in the community face-to-face. Since the intention was to recruit up to eight participants for each FGD, our interest was in the number of participants recruited for each FGD rather than how many people were approached and refused to participate. As is the case with typical qualitative studies, it was up to the community health mobilizer to decide who else to contact for recruitment as long as they ensured adequate representation of the different social network groups within the specific FGD allocated to a particular community.

Prior to each FGD, we conducted preliminary screening for eligibility based on age, and those who were below or above the age-category allocated to a particular community were excluded from the FGD. If any participants invited to participate in the FGD were excluded, this did not affect the overall participation in the FGD as long as the number of remaining participants was six or higher [36]. For adolescents aged 15–17 years (technically referred to as ‘minors’), we inquired to determine if they were ‘emancipated minors’ (e.g. whether or not they were married or lived an independent life away from their parents) and only those that were considered to be emancipated were included into the FGD, after consultations with the community health mobilizers.

### Data collection procedures and methods

Six FGDs were conducted to collect data from the three study communities using a pilot-tested FGD guide, designed exclusively for this study by the primary author (see Additional file 1). The guide was pilot-tested at Gaba landing site in Kampala, Uganda, which has the same characteristics as those of Kasensero, the study site; both being fishing communities. This helped us to refine the flow of questions in the guide as well as improve clarity in the questions asked to the participants.

Prior to starting the discussion, we collected socio-demographic data from each participant (age, education, marital status, prior HIV testing history, length of stay in the community, and occupation) using a socio-demographic form to be able to characterize the participants. The six FGDs were equally distributed throughout the three communities with two FGDs conducted at Kasensero landing site; two others conducted in Gwanda and the last two conducted in Kyebe, as summarized in Table 1. The FGDs were age- and sex-stratified so that two FGDs were conducted with adolescent boys and young men (15–24 years); two other FGDs were conducted with adolescent girls and young women (15–24 years) and the last two FGDs were conducted with adult men (25+ years). FGDs were conducted in pre-determined venues (e.g. town hall, room or other space at a health centre) that were selected by the community health mobilizer in each community while taking into consideration the ease with which participants would reach them and the extent to which the venues ensured privacy during the discussion. Participants were asked about their perceptions on community-based HIV self-testing (e.g. *If HIV self-test kits became freely available in the community, how comfortable would you be to obtain them from a member of your community?*), existing social network structures and how to distribute HIVST kits within these networks (e.g. *If we wanted to distribute HIV self-test kits to members of your groupings, how best would this be done? Who would lead the distribution exercise? What qualities would such a person have?*), and their perceptions and suggestions on social network-based, peer-led HIV self-testing (e.g. *How comfortable would people in this community be in obtaining HIV self-test kits from a peer-leader who has been trained to distribute HIV self-test kits?*). Participants were also asked questions on how best linkage to HIV care after a HIV-positive self-test could be improved (e.g. *How can more individuals be encouraged to seek confirmatory HIV testing at existing health facilities? How can linkage to HIV care among HIV-positive, self-tested individuals be improved?*)

FGDs were composed of between 7 and 8 participants drawn from different interest groups. Data were collected on a priori themes by two trained senior Social Scientists in the local language (*Luganda*) and were audio-recorded with permission from the participants. One of the Social Scientists (AN) served as the moderator while the other (SN) served as the note-taker. AN has a Bachelor of Social Sciences and a Master of Science in Population and Reproductive Health while SN has a Bachelor’s degree in Guidance and Counselling. Both AN and SC have over 5 years’ experience in qualitative research. FGDs took between 1 and 2 h. Participants were compensated for the time taken in participating in the FGDs (~US$2).

### Data analysis

All FGD interviews were transcribed verbatim and translated into the English language prior to analysis. Transcribed interview data were entered into a Microsoft Word processing document and later printed out in preparation for data analysis. Data were manually analysed deductively following the six phases of thematic framework analysis suggested by Braun and Clarke [37]. Initially, AN and SC transcribed the data and typed them into Microsoft Word documents. JKBM, AN and SC read through printed copies of the transcripts to ensure completeness of transcription while comparing them with the audio-recordings and the questions in the FGD guide. Once this process had been completed, AN and SC read through three of the six transcripts to generate codes based on nine a priori themes, i.e. i) willingness to use HIVST; ii) concerns about HIVST; iii) nature of support that people would need to properly perform HIVST and interpret their results correctly; iv) perceptions about peer-led HIVST; v) potential acceptability of a peer-led HIVST intervention in the community; vi) willingness for people to accept to receive HIV self-test kits from a peer-leader; vii) existing social network groups; viii) how HIV self-test kits could be distributed within existing groups, and ix) qualities of peer-leaders. This process generated a total of 32 codes.

Subsequently, JKBM and AN, separately, analysed the list of codes to decide which codes belonged to what themes, and if any codes belonged to more than one theme. Similar codes were merged to form one code. This process resulted in a final list of 20 codes that were used during the analysis. Each code represented a specific sub-theme within the main theme, and using these codes, we generated a list of issues that emerged around each sub-theme. Similar issues were grouped together to form categories. JKBM reviewed the final list of issues/categories generated during the analysis and linked them to the specific sub-themes to which they belonged. Each sub-theme was clearly linked to the primary theme to which it belonged. We then identified relevant quotations (representing views expressed across communities, sex and age-groups) that supported each issue/category within each sub-theme, and these quotations were used in the reporting of findings. Given the similarities in some of the quotations across sub-themes, we decided to group the initial nine a priori themes into four overarching themes, namely: i) perceptions on HIVST; ii) potential acceptability of peer-led HIV self-testing; iii) existing social network groups; and iv) how to distribute HIVST kits within existing social networks. This paper is based on the four themes that were determined through the above-mentioned iterative process. We followed the consolidated criteria for reporting qualitative studies (COREQ) in reporting the findings [38].

## Results

### Participants’ characteristics

Table 2 shows the socio-demographic characteristics of the 47 participants (31 men and 16 women) that participated in the FGDs. Overall, 68.1% were young people aged 15–24 years (*n* = 32); 19.1% (*n* = 9) were aged 25–34 years and 12.8% (*n* = 6) were aged 35+ years. Slightly more than half of the men (51.1%, *n* = 24) had attended school up to upper primary level of education (P5-P7) while 29.7% (*n* = 14) had attended school up to lower secondary education level (S1-S4), suggesting that 38 (80.8%) of the 47 participants had attended upper primary or post-primary education.

**Table 2:** Socio-demographic characteristics of FGD participants

Characteristic	Men [***N*** = 31] (n, %)	Women [***N*** = 16] (n, %)	Total (***N*** = 47) (n, %)
**Age-group**			
15–24	16 (51.6)	16 (100.0)	32 (68.1)
25–34	9 (29.0)	0 (0.0)	9 (19.1)
35+	6 (19.4)	0 (0.0)	6 (12.8)
**Education**			
None	1 (3.2)	1 (6.3)	2 (4.3)
Lower Primary (P1-P4)	5 (16.1)	1 (6.3)	6 (12.8)
Upper Primary (P5-P7)	17 (54.8)	7 (43.7)	24 (51.1)
Lower Secondary (S1-S4)	7 (22.6)	7 (43.7)	14 (29.7)
Upper Secondary (S5-S6)	1 (3.2)	0 (0.0)	1 (2.1)
**Prior HIV testing history**			
Ever tested for HIV	28 (90.3)	16 (100)	44 (93.6)
Never tested for HIV	3 (9.7)	0 (0.0)	3 (6.4)
**Length of stay in the community**			
< 1 year (less than 12 months)	0 (0.0)	3 (18.7)	3 (6.4)
1–2 years	1 (3.2)	2 (12.5)	3 (6.4)
3+ years	30 (96.8)	11 (68.8)	41 (87.2)
**Occupation**			
Fishing/Fishing-related activities	8 (25.8)	0 (0.0)	8 (17.0)
Other occupation	23 (74.2)	16 (100)	39 (83.0)

Almost all participants (93.6%, *n* = 44) had ever tested for HIV, although this proportion was slightly lower among men (90.3%, *n* = 28) than women (100%). Eighty-seven per cent of the participants had stayed in the community for three or more years, with a higher proportion of men reporting that they had stayed in the community for three or more years than females (men: 96.8%, *n* = 30; women: 68.8%, *n* = 11). By occupation, majority of the participants (83.0%, *n* = 39) were engaged in other occupations other than fishing or fishing-related activities.

### Perceptions about HIVST, potential acceptability of peer-led HIVST and distribution of HIVST kits in existing social network groups

As already noted, study findings were organized into four themes: a) perceptions about HIV self-testing as an additional HIV testing strategy in general; b) potential acceptability of a social network-based, peer-led HIV self-testing program; c) existing social networks in the community; and d) how to distribute HIV self-test kits to members in a social network. Each of these themes is presented in the following sub-sections.

- **Perceptions about HIVST as an additional HIV testing strategy**

In general, all participants had favourable attitudes towards HIVST with most participants reporting that HIVST is easy to perform; it offers an opportunity for busy people, who do not have the time to go to health facilities, to test for HIV and, most importantly, it takes away the need to incur travel costs to access health facility-based HIV testing services:

*According to me, the way I see myself and my fellow young people, they [people] will be willing to use HIV self-test kits because in most cases they are busy working so they do not get time to go to health centres for HIV testing and usually health centres are far requiring around UGX 2000-3000 [~US$ 0.5-0.8] for transport and yet I can spend a month without getting that money so if we are given those kits, it will help us to know our HIV status* (Adolescent boys and young men, 15–24 years, Gwanda)

While participants liked the fact that HIV self-testing can be conducted outside formal health facility settings, they were quick to point out that individuals who receive HIV-positive self-test results should go to the health facilities to have their results confirmed. However, given that people who self-test HIV-positive may not have the motivation to seek confirmatory HIV testing at existing health facilities without any additional encouragement, we asked participants what should be done to encourage self-tested HIV-positive individuals to seek confirmatory HIV testing. In response, participants suggested a need for the peer-leader who distributes the kits to educate their social network members about the importance of confirmatory HIV testing:

*Those who are given kits to distribute should counsel the people who go and get kits from them. They should tell them that if you test HIV positive, you should go to the health center. But if you give it to him/her without counselling him/her, he/she can see that she/he is HIV positive and stay back and fail to go to the health center to get medicine* (Adolescent girls and young women, 15–24 years, Gwanda)

Despite outlining the benefits of HIVST, there were mixed reactions as to whether one should conduct the test alone or in the presence of someone else that they trust. Those who preferred to test alone reasoned that testing in the presence of someone else could result in one’s results being known to other people, especially if the other person fails to keep the results secret. However, most of those who preferred to test in the presence of someone else reasoned that this person can help the self-tester to deal with unexpected results, especially in the event that one’s results turn out to be HIV-positive. The latter perspective reinforces the importance of providing pre- and post-test counselling to individuals who self-test for HIV:

*Considering what we have just been informed about HIV self-test kits, the whole process of HIV self-testing takes between 20-30 minutes better than all the other methods of HIV testing that we have been using. [However], how will you support yourself if you self-tested positive? Surely there is need of another trained person to be around so that you can be counselled* (Adult men, 25+ years, Kasensero)

Indeed, there were concerns that some people might fail to conduct the self-testing exercise correctly if they do it alone, and that HIV self-testing might cause some people to ‘hurt themselves’, i.e. commit suicide, if they self-tested HIV-positive without anyone to comfort them, as the following quotation illustrates.

*My concern is about a person ‘hurting’ himself if results of HIV self-test are positive. Because he will know his self-test results in a private place, there are higher chances of this person ‘hurting’ himself. So, if self-testing is done with someone around then such scenarios can be avoided* (Adult men, 25+ years, Kasensero)

There were also fears that people who self-test HIV-positive may be reluctant to seek confirmatory HIV testing or enrol into HIV care without the support of someone else especially if they initially thought they were HIV-negative but turned out to be HIV-positive.

*My fear is it’s very possible for someone to test HIV positive and [he/she] doesn’t go to the health facility to seek further HIV care especially if this person thought that he/she is HIV negative and the HIV self-test results show positive. Because he/she was not counselled enough, he/she may be reluctant going to the health facility to seek HIV confirmatory testing and HIV care* (Adult men, 25+ years, Kyebe)

When asked how these fears could be minimized, most participants suggested the need to train potential users of HIV self-test kits in how to use the kits; how to interpret their own HIV self-test results, and how to deal with the different types of HIV results before they are given the kits to use. Participants reasoned that such training would help users to self-test with ease and to deal with any associated consequences. There were also suggestions that, in addition to the training, users should be encouraged to seek HIV care if HIV-positive or to protect themselves from the risk of HIV infection if HIV-negative.

*Before you give him/her that kit, you should educate him/her on how he/she is going to use it to test. If he/she tests positive, you encourage him/her to go to the health center to get treatment. If he/she tests negative, you encourage him/her to protect her/himself that if he/she is going to have sex, he/she should use a condom* (Adolescent boys and young men, 15–24 years, Kyebe)

b) **Potential acceptability of peer-led HIVST**

Besides exploring people’s perceptions about HIVST in general, we sought participants’ views on peer-led HIVST; an approach where we intended to train local people in the community as ‘peer-leaders’ who would then distribute HIV self-test kits to members of their social networks. This approach is based on the assumption that people who belong to social networks know each other very well and can be easily reached with HIV testing services if HIV self-test kits were delivered to them by a member of their social network who has been trained in HIVST processes. Participants were particularly asked if such an approach would be acceptable to people in the community, and if so, we sought their opinions on how such an approach could be implemented in a fishing community. In response, most participants felt that the approach would be acceptable to people in the community ‘*because the person distributing HIV self-test kits could be my immediate neighbour which means he is near and easy to reach, I can easily access HIV self-test kits, go back to my home and do a self-test’*. Besides the ease of reaching the peer-leader, participants felt that use of a peer-led approach would make it easier for them to obtain the kits from their peer-leader at any time rather than go to the health facility which has opening and closing times:

*I can access them [kits] whenever I want unlike at the health center that closes at 2pm. But I can knock at that person’s door [any time] and tell him/her to give me the kit* (Adolescent girls and young women, 15–24 years, Kasensero)

Most participants reported that peer-led HIVST would help to improve people’s ability to test for HIV since the approach is private and ensures confidentiality of HIV test results. This is because the test is done in private and it’s upon the tester to disclose his/her results to their peer-leader or someone else. This approach was liked by the participants so much that some of them expressed the need to be selected as peer-leaders, reasoning that they can ably deliver the kits to their social network members and are approachable, social and trustworthy – key qualities necessary of a peer-leader:

*I suggest you give HIV self-test kits to me because now I have knowledge about them and I can also explain the test procedures to my group members and distribute the kits to them* (Adolescent boys and young men, 15–24 years, Gwanda)

*I would like to be part of the program, however, I am a fisher man and sometimes I fish from far distance from here, where by I spend more than two days in the lake yet you might need someone who is always on the mainland. … [but] yes, I would like to be part of the team distributing [kits] because I have all the qualities, I am approachable, social and trustworthy* (Adult men, 25+ years, Kasensero)

Since peer-led HIVST seemed acceptable to the participants, we asked them to identify which category of people they would be comfortable obtaining HIV self-test kits from in the community. In response, participants suggested that HIV self-test kits should be given to village health team (VHT) members or peer educators to distribute them to the people in the community.

*Most people fear going to health facilities, so I would prefer that they are given to VHTs or peer educators* (Adult men, 25+ years, Kyebe)

When asked about what qualities such people should have, participants mentioned that the community-based HIV self-test kits distributor should be a permanent resident of the area, someone who is not mobile (i.e. who can easily be found within the community at any time); educated to at least ordinary level of education (i.e. senior four); someone who easily relates with others, and most importantly, someone who can keep people’s secrets. Some participants suggested that peer-leaders should be trained in how to counsel other people, with emphasis put on how to keep people’s secrets:

*The person who will be given the kits should be trained first on how he/she should handle people so that he is able to keep people’s secrets so that he/she is not a rumourmonger who goes on telling people’s secrets to other people* (Adolescent boys and young men, 15–24 years, Kyebe)

*He/she should be friendly with everyone, trustworthy, keeps secrets, a resident who can be accessed any time. He/she should be educated and enlightened. He/she should be able to counsel others* (Adolescent girls and young women, 15–24 years, Gwanda)

Participants were concerned that if the community-based distributor was not good at keeping secrets, they could move in the community telling others how someone picked kits from them; something that could deter people from accepting the kits from them.

c) **Existing social networks in the community**

Since the peer-led HIVST program hinges on distribution of HIV self-test kits within social network groupings by a trained peer-leader, we asked participants, through free-listing, to tell us which social network groups exist within their community; and for any existing groups, to tell us if they are ‘big’ or ‘small’ groups (the definition of what constitutes ‘big’ and ‘small’ was left to the discretion of the participants), and whether or not they were male-only, female-only or of mixed gender. We also inquired of the possibility that some people could belong to several groups within the same community. In summary, a total of 21 social network groups were identified (Table 3); of these, 11 groups were male-only; four groups were female-only, while six groups had membership of both men and women.

**Table 3:** Existing social network groupings, stratified by membership gender

S/N	Social network grouping	Male members only	Female members only	Both male and female members
1	Boat pushers	√		
2	Drug user groups			√
3	*Boda-boda* cyclists [motorcycle taxis]	√		
4	Local brew drinkers	√		
5	Footballers	√		
6	Netballers		√	
7	Savings/cash-round groups			√
8	Sex workers		√	
9	Pool table players	√		
10	Talent show groups			√
11	Board game and playing card players	√		
12	Family planning groups		√	
13	MSM groups	√		
14	Religious groups			√
15	Fishermen	√		
16	Farmers groups			√
17	Betting clubs	√		
18	Unbound groups^a^			√
19	Bricklayers association	√		
20	Motorcycle mechanics group	√		
21	DREAMS^b^		√	

^a^Unbound is a non-profit organisation serving over 5000 beneficiaries in Uganda. They enrol children and youth into formal or informal education and help to unleash their potential to build an affluent, sustainable and equitable future. The organization supports groups of children consisting of between 15 and 20 members. In this paper, the term ‘unbound groups’ is used to refer to groups of youth that are supported by the organization within Kasensero fishing community

^b^DREAMS (Determined, Resilient, Empowered, AIDS-free, Mentored and Safe) is hereby used to refer to groups of adolescent girls and young women (15–24 years) who were enrolled in the DREAMS Project in Kasensero fishing community

In general, we found that some people belonged to more than one group in the same community. For example, a sex worker could be a member of a savings group and also a netballer. Similarly, *boda-boda* cyclists could be members of savings groups and also belong to a group of pool table players. However, members seemed to have loyalty to specific groups, and when asked which groups they belonged to, they cited one group in preference of the other(s). This information was necessary to inform the selection of peer-leaders and their social network members at the time of implementing the proposed peer-led HIVST intervention.

Our observation was that there were four biggest groups in the three study communities, namely: fishermen, savings/cash-round groups, sex workers and DREAMS. The group of fishermen was composed of men whose primary occupation is fishing; those who do the actual fishing in the lake, and other men involved in fishing-related activities, e.g. boat pushers. Boat pushers are groups of young men whose role is to push the boat from shallow waters to the mainland when fishermen return from a fishing expedition (so that the fish caught can be removed for sale). They also serve to push the boat back to the water when fishermen are setting off for another fishing expedition. Groups of fishermen can have about 500 members or even more.

*The fishermen and the boat porters/pushers have the biggest numbers of members because most adult men belong there. We are over 500 [members].* (Adult men, 25+ years, Kasensero)

On the other hand, groups of sex workers comprise all female sex workers who operate at Kasensero landing site under the leadership of a “dealer” or ‘pimp’ who acts as the ‘group manager’. Sex worker groups can have between 30 and 100 members. The ‘dealer’ is the person that men approach if they need to get a sex worker but her roles can stretch to resolving conflicts and arbitration between conflicting sex workers. Savings/cash-round groups can be composed of men, women or both men and women and exist to encourage members to save money for a defined purpose, usually under the leadership of a chairperson or group leader. Members contribute money to the group on a weekly basis. A member can borrow money from the group and return it with interest or the members can agree to receive back their savings on a rotational basis. Finally, the DREAMS group is composed of HIV-negative girls who have been enrolled into the DREAMS Project which is implemented by the Rakai Health Sciences Program. Girls enrolled in the DREAMS Project are encouraged to join existing savings groups or form new DREAMS-specific groups, and this is how the DREAMS savings group was created.

*“We have “DREAMS Tusitukiremu [Let’s Stand Together]” and “Little Angels”. Those groups help girls save money every week even if its five hundred, it teaches them how to save. A girl saves the money that she has every week starting from five hundred to ten thousand. After she has acquired skills in tailoring, that money helps her rent a house”* (Adolescent girls and young women, 15-24 years, Kasensero).

d) **How to distribute kits in existing social network groups**

After obtaining information about existing social network groups, we asked participants how best HIV self-test kits could be distributed within these networks. Most participants suggested a need to identify someone in the group, or a group leader, who could be trained in HIVST distribution processes and be given HIV self-test kits to take to his/her social network members.

*You should approach the boat pushers and tell them to select a leader, then the leader distributes the test kits to rest of the members* (Adult men, 15–24 years, Kasensero)

*There’s someone who heads the DREAMS program, it necessitates training that person because they can easily get to her since she is always around. They should train her so that she is the one that you meet and give the kits to* (Adolescent girls and young women, 15–24 years, Gwanda)

When asked if the person selected to distribute HIV self-test kits to members in a network should be male or female, on the whole, most participants felt that the sex of the distributor did not matter since it is all about obtaining the kits, and all one needs to do is ‘ … *to go and tell that person that I need a kit. He/she won’t know your results. He/she will just give you the kit and you go to your home to test*’, except that some young women preferred that the distributor be a man, because women may fail to keep secrets:

*“I would like to get it from a man, most women gossip a lot, they don’t keep quiet. It’s not easy for a man to give it to you and tell another man but with women, the moment you leave, she will tell someone”* (Adolescent girls and young women, 15–24 years, Gwanda)

One interesting finding, which was only identified in one group of young men, was the need to give out kits along with an identifying slip. These young men reasoned that the slips given along with the kits would be important in tracking the number of kits given out and the number of given kits that have been returned. We thought this was a wise idea and we eventually adopted it as part of the planned peer-led HIVST intervention:

*Let me [give] an example, we [are] here in this group that has a name, we need slips so that you give out a kit and a paper and after testing, you return the kit to the health worker. That kit should have that person’s name… those slips are going to help us note the number of kits that we have given out and the number that has been returned. We will know how many people have been able to know their status* (Adolescent boys and young men, 15–24 years, Kyebe)

### Suggestions on how to improve linkage to HIV care

Low linkage to HIV care after HIVST remains a critical issue given that not many people have the motivation to seek confirmatory HIV testing and/or linkage to HIV care services after an HIV-positive self-test. We sought participants’ views on the different approaches that can be used to improve linkage to HIV care following an HIV-positive self-test result. Some of the approaches explored included enrolment into community-based ART groups [where one person can collect HIV treatment from the health facility on behalf of other members in the group, and then another person does the same thing next time, and so on and so forth]; use of peer-leaders to deliver the initial ART package to their HIV-positive social network members; home-based delivery of the initial ART package by a health worker; and use of community-based HIV counsellors to deliver ART to HIV-positive individuals in the community.

In general, the use of peer-leaders to deliver ART to their HIV-positive social network members in the community was the most preferred approach when compared to other approaches. Participants argued that it would be important for peer-leaders to follow-up with their social network members (i.e. those that they gave kits to) to find out if some of them have self-tested HIV-positive. If they find that some of their members self-tested HIV-positive, they should encourage them to seek confirmatory HIV testing and, if confirmed to be HIV-positive, link to HIV care as soon as their HIV-positive status has been confirmed.

*A peer educator can intervene and take HIV treatment to the homes of the self-tested individuals… [The use of a peer-leader] could be effective because the peer leader can reach his colleague who self-tested HIV positive without informing others… it is better if they get linked through the peer leader who distributed the kits* (Adult men, 25+ years, Kyebe)

*Those who are given kits to distribute should counsel the people who [got] kits from them. They should tell them that if you test HIV positive, you should go to the health center. But if you give it to him/her without counselling him/her, he/she can see that she/he is HIV positive and stay back and fail to go to the health center to get medicine* (Adolescent girls and young women, 15–24 years, Gwanda)

There were divided opinions on the ability of the other approaches to improve linkage to HIV care among self-testing HIV-positive individuals. For instance, home-based delivery of ART was not found feasible because ‘ … *this individual [the person using the kit] did a self-test and the results are known to them alone*’ (adult men, 25+ years, Kyebe). Thus, while some participants thought that home-based delivery of ART could help HIV-positive people to link to HIV care, other participants were concerned that if the community got to know that a health worker usually visits their home to deliver ART services, that would make them uncomfortable.

*“In my opinion it’s a good [idea] because no transport cost is involved by self-tested individuals. However, if community members become aware that a medical worker always visits a certain person in the village, they become suspicious that the person is HIV positive which may make the one [who] obtains ARVs from his/ her home uncomfortable”* (Adolescent boys and young men, 15–24 years, Gwanda)

Similarly, community ART groups were not found appropriate because ‘…*this person has not yet identified himself with them [members of community-based ART groups], unless you as the health workers talk to this person about the existing ART groups in the communities* (Adult men, 25+ years, Kyebe). In another group, participants had this to say: ‘*It [use of community ART groups] is not good, people will know that group is for those who are HIV positive. People will be afraid of it*’ (Adolescent boys and young men, 15-24 years, Kyebe). However, other participants thought that use of community ART groups could be a good strategy since all members are HIV-positive and these could counsel each other: ‘*That one [use of a community-based ART group] is good. You can’t gossip about me since all of us are swallowing medicine*’ (Adolescent girls and young women, 15–24 years, Gwanda). This latter perception was also echoed by adolescent boys and young men (15–24 years) in Gwanda: ‘*In my opinion that’s a good idea because no one who is likely to rumourmonger about the other [since] all of them are HIV positive. It will also encourage a new self-tested HIV-positive individual to enrol into HIV care because the ART group mates will share encouraging experiences with him/her*’.

The use of community-based HIV counsellors to deliver ART to the HIV-positive individuals in the community received mixed reactions with some participants arguing that it could help to improve linkage to HIV care since the counsellor can counsel the individual into starting HIV treatment. However, other participants thought that if counsellors are not trustworthy, they may end up telling other people in the community which might discourage people from accepting them in their homes.

*May be if the counsellors are trustworthy but he/she might give it you and then if she finds someone on her way, she will tell him/her that I was at this person’s place, I had gone to take for her/him medicine. Or you can hear people in the bar saying that this person’s daughter or this person’s wife swallows medicine, I just took it there* (Adolescent girls and young women, 15-24 years, Gwanda).

## Discussion

Our findings from this formative study of a peer-led HIVST intervention suggest three pertinent issues that can inform future community-based HIVST programs: a) Peer-led HIVST is acceptable among community residents who believe that it is easier to obtain kits from someone that they live with and who they can approach any time to get the kits; b) existing social network groupings have leaders that can be trained as peer-leaders (or new individuals can be identified within the groups and trained) to distribute HIV self-test kits within their social networks; and c) selected peer-leaders should be approachable, resident in the same community as their social network members and be able to keep secrets. The finding that peer-led HIVST was acceptable was important for the design of the planned peer-led HIVST intervention in which we planned to work with selected local people, train them as peer-leaders, and ask them to distribute HIV self-test kits within their networks.

Previous research on peer-led HIVST interventions suggests that the use of peer-leaders can be an important attribute in reaching out to members of social networks that may not access conventional HIV services [27, 28]. In a pilot trial of the peer-based distribution of HIV self-test kits among fishermen in Buliisa, Uganda, Choko, Nanfuka, Birungi et al. [27] found that 82% of men who were reached with HIV self-test kits by their peers accepted to self-test for HIV. In another study to assess the effect of peer distribution of HIV self-test kits to men who have sex with men on HIV testing uptake among men with undiagnosed HIV infection in Uganda, Okoboi, Lazarus, Castelnuovo, et al. [28] found that 95% of the MSM who were reached with fellow MSM with HIV self-test kits accepted to test for HIV. Evidence from prior studies [39, 40], along with our findings, suggest that the use of a peer-led HIVST approach can help to improve uptake of HIV testing services in populations that cannot easily access conventional HIV services either due to their mobility, physical location, sexual orientation or other barriers.

Although peer-led HIVST seemed to be an acceptable strategy to reach people with HIVST kits in this setting, some participants were concerned that testing alone with a HIVST, without any pre-test counselling provided by a professional healthcare worker, might result into dire consequences, including fears that some HIV-positive people might commit suicide after failing to cope with their new HIV-positive diagnosis. These concerns have been documented in previous studies, especially in populations where HIVST kits have not yet been introduced [41, 42]. However, studies conducted in populations where kits have been introduced have not confirmed these fears. There are no studies reporting suicide after performing HIVST [18, 43, 44], although a few social harms have been reported. For instance, Kumwenda, Johnson, Choko, et al. [45] identified one (1) serious harm (predominantly break-up of newly identified married HIV sero-discordant couples) per 10,000 HIVST kits distributed. There were also a few cases of blame and frustration among newly diagnosed concordant HIV-positive couples but rarely did these episodes lead to any serious harm. Although no serious harms have happened as a result of HIVST promotion, our observations point to the need to refer HIV-positive individuals to existing referral mechanisms for further management and enrolment into HIV care. As discussed below, the use of peer-leaders can be a useful link between the participant testing HIV-positive and the known sources of care.

Participants called for the selection of peer-leaders who are trustworthy and who can keep people’s secrets. Previous studies, conducted in other settings, have found that people tend to fear being tested for HIV by health workers for fear that they might tell others about their results [46, 47]. This apparent lack of trust among providers was the very reason that some participants did not think that the use of community-based HIV counsellors could improve linkage to HIV care after HIV self-testing. Similar sentiments were raised with regard to home-based initiation of ART by a health provider, with some participants fearing that if the health provider makes frequent visits to one’s household, the community might begin to suspect that the person being visited could be HIV-positive. The issue of keeping one’s results confidential is related to the high levels of stigma that still exist in the Ugandan community [48], including in this hyperendemic community, and points to the need for careful selection of peer-leaders who are trusted within their respective social network groups.

Linkage to HIV care remains a critical challenge across HIVST studies [49, 50]. In a recent implementation study of oral and blood-based HIV self-testing and linkage to care among men in rural and peri-urban KwaZulu-Natal, South Africa, Shapiro, van Heerden, Krows, et al. [49] found 32% of HIV-positive men did not link to HIV care after 2 months of their initial HIVST positive result citing lack of time to go to clinic, having to be at work during the hours that the clinic is open, and being concerned about long wait times at the HIV clinic. These findings suggest a need for interventions that can reach men at their work place, or those that can reduce the need to go to the HIV clinic for HIV care. Our findings show that use of peer-leaders can be an acceptable strategy to improve linkage to HIV care among those testing HIV-positive, especially if the peer-leaders have been trained to offer basic post-test support to their peers and to demonstrate the benefits of timely linkage to HIV care among their social network members. Some participants suggested that home-delivery of ART by peer-leaders to their social network members can help to improve linkage to HIV care since it takes away the need to incur transport costs to go to a health facility. A study that assessed the effect of offering same-day ART vs. usual health facility referral during home-based HIV testing on linkage to care and viral suppression among adults with HIV in Lesotho demonstrated that immediate ART initiation after community-based HIV testing increased linkage to care at 3 months compared to referral to clinic facilities to initiate treatment [51], and a study of optional home-based initiation of ART after HIV self-testing in Malawi found that provision of optional home-based initiation of ART improved ART initiation in the home-based arm than in the facility-based group [52]. Thus, future studies may find it prudent to explore the possibility of using peer-leaders to deliver ART to their social network members, given adequate support and a well-functioning referral mechanism to deal with more complicated cases.

We found that up to 21 social network groups existed in the study communities although there was a high possibility that these were not distinct groups since membership to several groups was possible. Nevertheless, the finding that more organized groups such as those of sex workers, savings groups and fishermen had identified someone as their leader was an important finding that helped to inform the design of the planned peer-led HIV self-testing intervention. Social network-based HIV testing has been recommended as a key strategy to reach key populations, including those that are currently missed by conventional HIV testing programs [53]. Thus, besides providing information to inform the design of our planned intervention, study findings lend credence to the implementation of social network-based interventions through identification of locally existing social groupings from whom peer-leaders can be identified and trained in how to distribute HIV self-test kits to members of their social network. Although prior efforts have been made to promote HIV self-testing among key populations such as sex workers [54] and truck drivers [55], previous efforts have not focused on using a social network-based strategy to reach members of these key populations. Our research advances the need to identify locally existing groups, and within these groups, select a “peer-leader” who can be trained to distribute HIVST kits within their social networks.

This study had a number of limitations. The data were collected using only one method, focus group discussions, thereby affecting our ability to triangulate the findings using different data collection methods. However, we ensured that the participants were drawn from different population groups in the three study communities; thereby increasing the likelihood that the views expressed by these participants reflected practical realities in the different communities. However, since the focus group discussions were conducted before the social network groups were identified, it is likely that we did not get a fair representation of the different groups that existed within the community at the time, thereby affecting the generalizability of findings to all the groups in the fishing community. It is also important to note that for adolescents under 18 years of age, we only enrolled those that were ‘emancipated’. It is likely that the challenges experienced in accessing HIV testing and/or linkage to HIV care services (if found to be HIV-positive) might differ between adolescents who live on their own (or who are married) and those that still live with their parents. Also, although this study was planned as part of a network-based study, we did not go into the full length and breadth of social network analysis, since this study was intended to identify social network groups for purposes of selecting peer-leaders. For that reason, the data collected about the groups was only that which was needed to inform the design of the intervention and no further attempt was made to collect additional social network data that would be collected in a typical social network study. Finally, given that study participants had higher rates of prior HIV testing, it is likely that their views might be different from the views of those with lower rates of prior HIV testing. Thus, our findings should be interpreted with this observation in mind. Future studies should be conducted in areas of low or limited HIV testing access in order to obtain insights about peer-led HIVST from settings with limited access to services.

Despite these limitations, we believe that the study findings provide the impetus for the use of peer-led HIVST programs to target hard-to-reach populations, including fishing communities, and indeed, the findings provided insight into the nature of existing social network groupings in Kasensero fishing community as well as the qualities that selected peer-leaders should have. Although HIV self-test kits were available at the existing health facilities, these kits were only distributed to those who accessed health services at these health facilities but they were not available for community distribution. Thus, this is the first study to explore the perceptions about and opportunities for implementation of a community-based, peer-led HIVST distribution intervention in this fishing community. The favourable attitudes towards peer-led HIVST model and the views expressed by the participants, including suggestions on how to implement this program, suggest that community-based distribution of HIVST is a welcome intervention in Kasensero fishing community.

## Conclusion

Our study found that favourable attitudes towards the implementation of a peer-led HIVST intervention in the fishing community. Participants reported that such an intervention could increase uptake of HIV testing in the community given that people can easily access HIV self-test kits from their peer-leaders who live in the same community without the need to travel to the health facilities. Our findings show that peer-leaders should have defined qualities (e.g. able to keep secrets) if the peer-led HIVST program is to be successful. These findings suggest that implementation of peer-led HIVST interventions may increase uptake of HIV testing services among under-served populations who belong to existing social network groupings.

## Supplementary information

**Additional file 1.** Study tool. Focus Group Discussion Guide.

## Data Availability

The datasets generated and/or analysed during the current study are not publicly available due to individual privacy concerns (given that this is a qualitative study where it is completely difficult to anonymize the data) but are available from the corresponding author on reasonable request.

## Abbreviations

AIDS: Acquired Immunodeficiency Virus
ART: Antiretroviral Therapy
COREQ: Consolidated Criteria for Reporting Qualitative Studies
DREAMS: Determined, Resilient, Empowered, AIDS-free, Mentored, Safe
FGD: Focus Group Discussion
HIV: Human Immunodeficiency Virus
HIVST: HIV self-testing
MSM: Men who have sex with men
P1-P4: Primary one to primary four (level of education)
P5-P7: Primary five to primary seven (level of education)
SSA: Sub-Saharan Africa
S1-S4: Senior one to senior four (level of education)
S5-S6: Senior five to senior six (level of education)
UGX: Uganda Shillings
UNAIDS: Joint United Nations Program on HIV/AIDS
UPHIA: Uganda Population-based HIV Impact Asssessment
US: United States
