# Supplementary material for: Formative research to inform the development of a peer-led HIV self-testing intervention to improve HIV testing uptake and linkage to HIV care among adolescents, young people and adult men in Kasensero fishing community, Rakai, Uganda: a qualitative study
Source: BMC Public Health. 2020 Oct 20;20:1582. doi: 10.1186/s12889-020-09714-1 (PMC7576713; doi:10.1186/s12889-020-09714-1)
Supplement: Supplementary file 1 — Additional file 1. Study tool. Focus Group Discussion Guide. [file 12889_2020_9714_MOESM1_ESM.doc]

**FOCUS GROUP DISCUSSION GUIDE**

**[QUALFORM – FGD/HIVST/01]**

**Study Title: Implementing a Network-based, Peer-led HIV Self-testing Intervention to Improve HIV Testing and Linkage to HIV Care among Young People and Adult Men in Kasensero Fishing Community, Rakai District**

**Section A: Beginning Questions**

1. How easy is it for people to access HIV testing services in this community?
2. If people test HIV-positive, what challenges do such people face in accessing HIV treatment? How can these challenges be minimized?

**Section B: Perceptions of HIV Self-testing**

HIV self-testing is a way of seeing if you have contracted HIV by conducting the test yourself, instead of having to see a doctor and ask for a test. Special kits let you swab your gums to collect some saliva, and then test the saliva to see if there’s any sign of HIV in your body. You can do this entirely by yourself in the privacy of your own home, and it takes about 20-30 minutes to complete.

1. What are your general impressions about HIV self-testing? How willing will people in your community be to use HIV self-test kits to test for HIV? Please explain your response.
2. What kind of support would people need before they conducted the self-test? What kind of support would they need after conducting the self-test?
3. In your opinion, would it be better if people self-tested alone or if they self-tested with someone around them? Please explain your response.
4. In your opinion, should HIV self-test kits be given to all people in the community or to selected people? If you had free HIV self-test kits to distribute in the community: a) which people or groups of people would you prioritize? b) which people would you deny kits? Please explain your response.
5. Should HIV self-test kits be denied to young people who are not yet of age (i.e. below 18 years)? If young people were given HIV self-test kits, how should they be supported to use them to test for HIV?
6. If HIV self-tests became available in this community, where would people be comfortable to obtain them? Probe for: health facility, VHT, friend, peer educator, Beach Management Unit, etc.
7. What fears or concerns do you have in general regarding HIV self-testing?

**Section C: Perceptions of community-based HIV self-testing distribution**

1. If HIV self-test kits became freely in the community, how comfortable would you be to obtain them from a member of your community?
2. Which kinds of people would you be comfortable obtaining kits from? What qualities should they have?
3. What challenges do you envisage with community-based distribution of HIV self-test kits? How can these challenges be minimized?
4. How can confidentiality be ensured in a setting where people obtain kits from a member of their community rather than at the health facility?

**Section D: Social Network Structures**

1. What social groupings of: a) young people and b) adult men exist in this community?
2. How many members do such groupings have? How do members join these groupings? Who is eligible to join the grouping? How often do members exit the groupings? *Probe for membership aspects for groups of young men and groups of adult men separately.*
3. How often do such groupings meet? Where do they meet? How are their meetings organized?
4. What benefits do: a) young people and b) adult men obtain from belonging to such groupings?
5. How many people in this meeting belong to any social network groupings in this community? Please tell me more about the groupings that you belong to.
6. If we wanted to distribute HIV self-test kits to members of your groupings, how best would this be done? Who would lead the distribution exercise? What qualities would such a person have?

**Section E: Perceptions and Suggestions on Network-based, Peer-led HIV Self-testing**

[Name hidden] (hereafter referred to as the Research Team), with funding from Fogarty International Center and Africa Research Excellence Fund, intends to implement a program in which people in this community will receive free HIV self-test kits to use to test for HIV and learn about their HIV status outside formal health facilities. The kits will be distributed by local people – who are members of this community. These individuals will be selected by the Research Team in consultation with community leaders, and they will be trained in how to use HIV self-test kits as well as read and interpret HIV self-test results. Each trained person will recommend up to 20 people to the Research Team (those recommended should be known to the person recommending them, including friends, work-mates, family members, or members of existing social groups) to be considered for receiving free HIV self-test kits. Because we don’t have enough kits at the start of this program, only 10 of the 20 people recommended, who are eligible for inclusion in the research, will receive the kits. Each of the 10 people who will receive the HIV self-test kits will be trained in how to use them and how to interpret their own HIV self-test results. Individuals that will test HIV-positive will be encouraged to seek **confirmatory HIV testing** at one of two participating health facilities, and if confirmed to be HIV-positive, they will be linked to HIV care immediately as per the Ministry of Health’s Test and Treat policy guidelines. The purpose of the program is to improve HIV testing and linkage to HIV care among young people (15-24 years) and adult men (25+ years) living in Kasensero fishing community by introducing a method of HIV testing that can be done outside formal health facilities.

1. What are your initial thoughts about such a program? If you had the opportunity, would you have liked to be part of this program? Why or why not?
2. How comfortable would people in this community be in obtaining HIV self-test kits from a peer-leader who has been trained to distribute HIV self-test kits?
3. What benefits do you envisage in implementing such a program in this community? How can these benefits be maximized?
4. What challenges do you envisage in the implementation of this program in this community? How can these challenges be minimized?
5. If you were asked to select someone who should be trained to distribute HIV self-test kits to young people aged 15-17 years in this community, what qualities would you look for?
6. If you were asked to select someone who should be trained to distribute HIV self-test kits to young people aged 18-24 years in this community, what qualities would you look for?
7. If you were asked to select someone who should be trained to distribute HIV self-test kits to adult men (25 years or older) in this community, what qualities would you look for?
8. How would confidentiality be ensured in a program that distributes HIV self-test kits through trained local distributors?
9. If you were one of those individuals who have been selected to receive HIV self-test kits from a trained local HIV self-test kits distributor, would you prefer that you receive the kit from a same-sex distributor or would this not matter? Where (venues) would you prefer to receive the kits from? Why those particular places?
10. Individuals who self-test HIV-positive are usually advised to seek confirmatory HIV testing at a government health facility. In your opinion, would HIV-positive self-tested individuals accept to seek confirmatory HIV testing at existing health facilities? How can more individuals be encouraged to seek confirmatory HIV testing at existing health facilities?
11. In previous studies, many individuals who self-tested HIV-positive did not enroll into HIV care at existing health facilities. In your opinion, what reasons may stop HIV-positive, self-tested individuals from enrolling into HIV care? How can linkage to HIV care among HIV-positive, self-tested individuals be improved? *Probe for: optional home-based ART initiation; linkage to care through community-based HIV counsellors/expert clients in the community; use of community ART groups to link people to care; and linkage to care through peer-leaders*
12. In general, how would this kind of program be improved to reach more people in the fishing communities?

**THANK YOU FOR YOUR TIME**
